# Brain reactions to the use of sensorized hand prosthesis in amputees

**DOI:** 10.1002/brb3.1734

**Published:** 2020-09-19

**Authors:** Giuseppe Granata, Riccardo Di Iorio, Francesca Miraglia, Massimo Caulo, Francesco Iodice, Fabrizio Vecchio, Giacomo Valle, Ivo Strauss, Edoardo D'anna, Francesco Iberite, Liverana Lauretti, Eduardo Fernandez, Roberto Romanello, Francesco M. Petrini, Stanisa Raspopovic, Silvestro Micera, Paolo M. Rossini

**Affiliations:** ^1^ Institute of Neurology Fondazione Policlinico A. Gemelli‐IRCCS Roma Italy; ^2^ Department of Neuroscience & Rehabilitation IRCCS San Raffaele Pisana Roma Italy; ^3^ Institute of Neurology Catholic University of The Sacred Heart Roma Italy; ^4^ Department of Neuroscience and Imaging and ITAB‐Institute of Advanced Biomedical Technologies University G. d'Annunzio Chieti Italy; ^5^ The BioRobotics Institute and Department of Excellence in Robotics and AI Scuola Superiore Sant'Anna Pisa Italy; ^6^ Center for Neuroprosthetics and Institute of Bioengineering School of Engineering École Polytechnique Fédérale de Lausanne (EPFL) Lausanne Switzerland; ^7^ Laboratory for Neuroengineering Department of Health Sciences and Technology Institute for Robotics and Intelligent Systems ETH Zürich Zürich Switzerland; ^8^ Institute of Neurosurgery Fondazione Policlinico A. Gemelli‐IRCCS Roma Italy

**Keywords:** advanced biotechnologies, brain function, hand prosthesis, personalized medicine

## Abstract

**Objective:**

We investigated for the first time the presence of chronic changes in the functional organization of sensorimotor brain areas induced by prolonged training with a bidirectional hand prosthesis.

**Methods:**

A multimodal neurophysiological and neuroimaging evaluation of brain functional changes occurring during training in five consecutive amputees participating to experimental trials with robotic hands over a period of 10 years was carried out. In particular, modifications to the functional anatomy of sensorimotor brain areas under resting conditions were explored in order to check for eventual changes with respect to baseline.

**Results:**

Full evidence is provided to demonstrate brain functional changes, and some of them in both the hemispheres and others restricted to the hemisphere contralateral to the amputation/prosthetic hand.

**Conclusions:**

The study describes a unique experimental experience showing that brain reactions to the prolonged use of an artificial hand can be tracked for a tailored approach to a fully embedded artificial upper limb for future chronic uses in daily activities.

## Graphical abstract

Recent technological advancements have allowed for a bidirectional connection of the prosthetic hand via intraneural electrodes in stump nerve trunks. No reports are available so far on modifications in brain function accompanying the use of a bidirectional hand prosthesis. We performed a multimodal neurophysiological and neuroimaging evaluation of brain functional changes occurring during training with a bidirectional hand prosthesis in five consecutive patients, and demonstrating brain functional changes related to the use of the prosthetic hand.

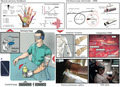

## 1 INTRODUCTION

The hand is a body segment of extraordinary importance as it is used to interact with the peri‐personal environment in all the pivotal daily activities; its loss causes severe physical and psychological deficits.

Hand amputation is followed by a cascade of plastic changes in motor and somatosensory pathways and relays in the “orphan” districts of the central nervous system (CNS) connected to the amputated part. The greater availability of adequate tools for evaluating the cerebral cortex makes this district the most studied of nervous system. The first described feature of cortical plastic reorganization following limb amputations is the invasion of the “deafferented” cortex by the cortical representation of adjacent body districts in the primary sensory and motor cortices (Merzenich, 1998; Wrigley et al., 2009). Several recent experimental studies and observations in human amputees clearly demonstrated the persistence of a certain degree of functionality of the somatosensory and motor cortices corresponding to the amputated part, even several years following amputation (Granata et al., 2018; Makin, Filippini, et al., 2015; Makin et al., 2013; Makin, Scholz, Henderson Slater, Johansen‐Berg, & Tracey, 2015; Raspopovic et al., 2014; Rossini et al., 2010). Both the cortical reorganization and the persistance of the original functional topography seem to contribute to the presence and severity of the phantom limb pain (PLP) (Flor et al., 1998; Flor et al., 1999; Vaso et al., 2014). Moreover, recent researches pointed out the presence of plastic changes occurring at the whole sensorimotor network after hand amputation (Serino et al., 2017; Valyear, Mattos, Philip, Kaufman, & Frey, 2019), demonstrating an impoverishment of the network used for grasping in terms of brain areas activated during the task, intensity of activation and interareas connectivity.

The future of prosthetics is moving toward the use of a new generation of devices giving to the amputee the ongoing possibility of performing complex movements and receiving real‐time somatosensory feedback in an open loop (Raspopovic et al., 2014; Schiefer, Tan, Sidek, & Tyler, 2016; Tan, Schiefer, Keith, Anderson, & Tyler, 2015; Tan et al., 2014; Tyler, 2015). A limited literature is available on plastic brain changes accompanying the use of artificial upper limbs, and—so far—no reports have been published concerning brain reactions to the use of sensorized hand prosthesis(Chen, Yao, Kuiken, & Dewald, 2013; Serino et al., 2017; Yao & He, 2001).

This study reports on a multimodal evaluation of cortical changes occurring during bidirectional hand prosthesis training of variable duration (4–36 weeks), in a series of 5 amputees over a period of 10 years. Primary endpoint was to investigate the presence of chronic changes in the functional organization of sensorimotor brain areas due to amputation at baseline and to follow up eventual modifications induced by training with a hand prosthesis and sensory feedback.

## 2 SUBJECTS, MATERIALS, AND METHODS

The hand prosthesis, the stimulation unit and the dedicated software were the output of several consecutive European Union and Italian Ministry of Health funded projects involving research teams from different EU countries; some of them were described in dedicated previous papers (Petrini et al., 2019; Raspopovic et al., 2014; Rossini et al., 2010). All the human experiments reported here were carried out in Italy after approval by the local Ethics Committee and by the Italian Ministry of Health. Written informed consent was provided by the patients before trial initiation.

### 2.1 Patients

The results from five consecutive left transradial amputees are included in this report.

*Patient 1*: 26‐year‐old right‐handed male with a left transradial amputation that occurred in 2007. At the time of the study (2.5 years after amputation), the patient was not using any kind of prosthesis and was affected by mild phantom limb pain (scored as 4 according to the VAS).

*Patient 2*: 34‐year‐old male with a traumatic transradial (proximal third of the forearm) amputation of the left arm in January 2004. At the time of the trial (3 years after amputation), the patient suffered from severe phantom limb syndrome, scored as 9 on the VAS.

*Patient 3*: 37‐year‐old male with a traumatic transradial (proximal third of the forearm) amputation of the left arm that occurred 2 years before the trial. The patient, before the amputation, was left‐handed, and at the time of the trial, the patient was affected by severe PLP (score of 8 on the VAS).

*Patient 4*: 48‐year‐old female with a traumatic transradial (distal third of the forearm) amputation of the left arm that occurred 23 years before trial. She was free from phantom pain and was in chronic treatment with antidepressant drugs at a low dosage because of mild depression.

*Patient 5*: 53‐year‐old female with left traumatic transradial (proximal third of the forearm) amputation that occurred 1 year and 6 months before the trial. She suffered from mild phantom limb pain and very frequent nonpainful phantom sensation (score of around 4 on the VAS), mainly located on the ulnar side of the phantom hand.

**FIGURE 1:**
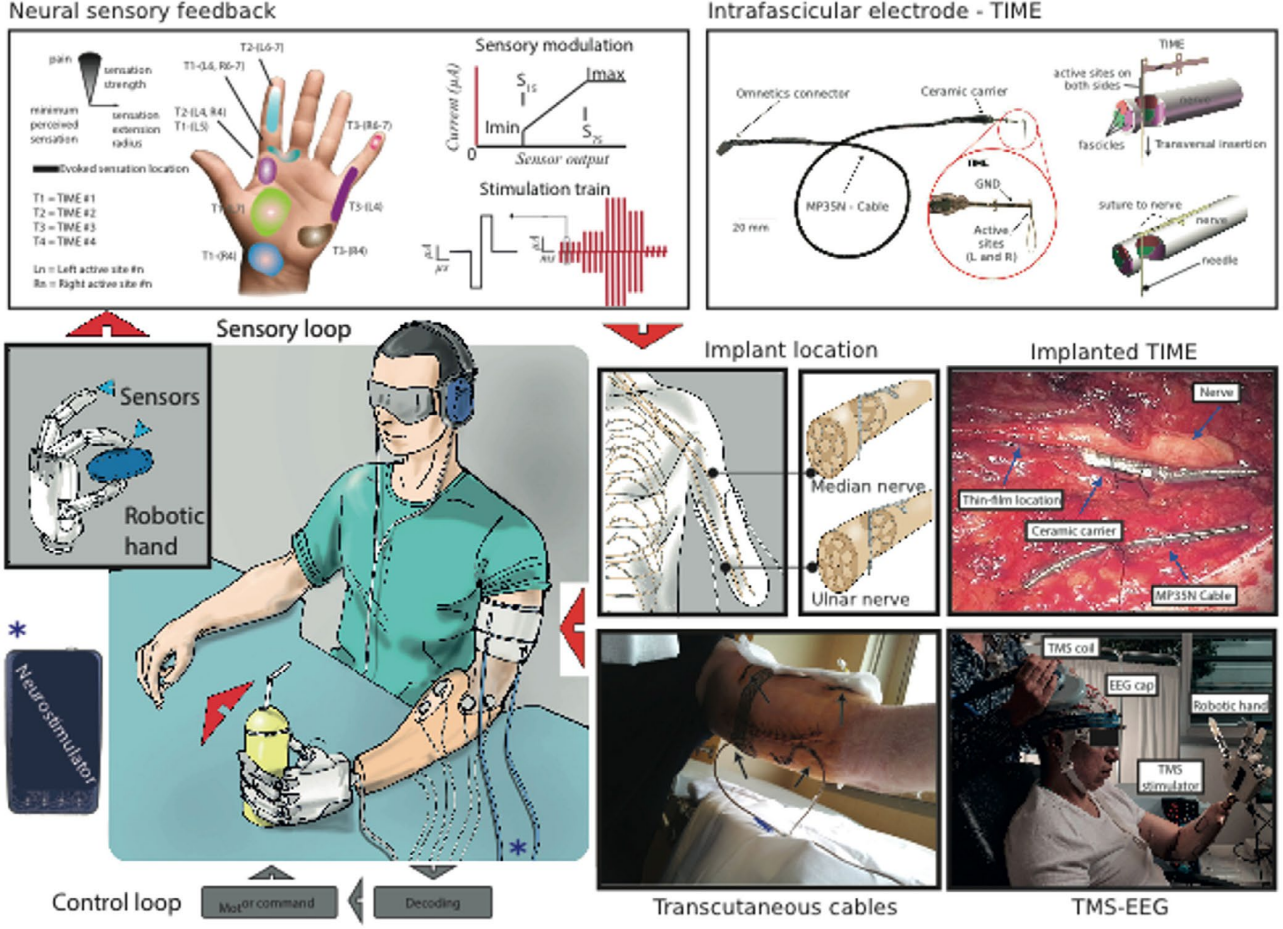
Left lower and upper panels: the prosthetic system (the sensorized prosthesis, the EMG electrodes for the muscular activity recording, the electrical stimulator, and the TIME electrodes implanted on the nerves) and the functioning of the whole system during a grasp task (EMG from the stump muscles during voluntary contraction was recorded with surface electrodes, used to decode efferent activity, and translated into orders for movement of the hand prosthesis; the contact and force production of the prosthetic fingers against resistance triggered a rapid cascade of events within a time window of a few tens of milliseconds, generating a real‐time perception of a sensation from the phantom hand/fingers, thanks to the proportional intensity of electrical stimulation of the stump nerves with the TIME connected to the stimulator). Middle lower panels: localization of TIME electrodes into the median and the ulnar nerves above the elbow, the TIME electrode inserted in the nerve during surgery, and the transcutaneous electrodes available for the connection with the stimulator at the end of surgery. Upper right panel: structure of the TIME electrodes and procedure of their insertion via a guiding needle. Right lower panel: setting of TMS‐EEG performed with the neuronavigation system for exact positioning and repositioning

The artificial hand model utilized for *Patient 1* was too heavy to be wearable and was experimented under remote control on the laboratory table; such condition decreased the mechanical/electronic noise to a level that allowed reliable recordings of the motor output signals from the motor nerve fibers, which were translated into movement commands to the hand prosthesis (Rossini et al., 2010). The remaining patients were using a more advanced model which was directly connected to the socket on the stump. This did not allow any direct intraneural recording from motor nerve fibers of the stump due to the electronic/mechanical noise. Therefore, surface electromyographic recordings were employed to drive the motor commands to the prosthesis (Figure 1, see Raspopovic et coll. 2014 (Raspopovic et al., 2014)) as fully described in previous technical publications (Raspopovic et al., 2014; Rossini et al., 2010).

*Patients 1* and *2* used the system for 1 month with daily trials. *Patient 3* used the system almost daily for 9 months while *Patients 4* and *5* used the system for 5 months, almost daily in the first 2 months and 2 or 3 days a week afterward. During the training period, each patient performed different tasks aiming to recognize the physical properties of objects (including shape, consistency, and texture), to perform a variety of simple and complex movements, and to produce different levels of force and pressure. Moreover, many hours of nerve stimulation were devoted to psychophysical topographical mapping of the sensory input and to validate its stability in time.

The patients underwent a multimodal neurophysiological and neuroimaging examination, including electroencephalography (EEG), the recording of EEG responses to transcranial magnetic stimulation of the motor cortex (TMS‐EEG), and structural and functional magnetic resonance imaging (MRI and fMRI).

Table 1 summarizes the type and timing of each technology used in individual patients.

**TABLE 1:** Characteristics of patients, summary, and timing of tests

	Time of amputation	VAS baseline	VAS T1	Level of amputation	Previous Prosthesis	EEG	TMS‐EEG	MRI/fMRI
Pt. 1	2 years and 6 months	4	2	Transradial	No		2 times: baseline and T1	
Pt. 2	3 years	9	4	Transradial	Myoelectric	2 times: baseline and T1		
Pt. 3	2 years	8	4	Transradial	Cosmetic	4 times: baseline, 9th week, 18th week, end of trial (T1)	3 times: baseline, 18th week, end of trial (T1)	3 times: baseline, 18th week, end of trial (T1)
Pt. 4	23 years	0	0	Transradial	No	3 times: baseline, 8th week, end of the trial (T1)	2 times; baseline and T1	
Pt. 5	1 year and 6 months	4	4	Transradial	Cosmetic	2 times: baseline and T1	2 times: baseline and T1	2 times: baseline and T1

Abbreviations: EEG, electroencephalography; fMRI, functional magnetic resonance imaging; MRI, magnetic resonance imaging; TMS‐EEG, TMS performed during EEG recording.

### 2.2 Procedures to analyzed brain reactions

*Transcranial magnetic stimulation EEG (TMS‐EEG)* was performed using a Magstim Rapid2 Stimulator (Magstim Company Limited) in order to stimulate the motor cortex with simultaneous EEG recording via a BrainAmp DC (Brain Product GmbH) device with 57 electrodes positioned on standardized scalp sites according to the augmented 10–20 international system. Three additional channels were simultaneously acquired for the electro‐oculogram and the electrocardiogram. Other four electrodes were used for a bipolar recording of the motor‐evoked potential (MEP). For the magnetic stimulation, a Magstim 200 magnetic stimulator connected to an 80‐mm figure‐of‐eight coil was used. The stimulation was performed by positioning the virtual cathode of the coil over the site of the scalp to be stimulated with the holder oriented at a 45° angle with respect to the approximate direction of the central sulcus. MEPs were recorded from the biceps and/or extensor and flexors muscles of the forearm on both sides via pairs of Ag/AgCl surface electrodes placed over the belly of the muscle. The resting motor threshold (RMT) was defined—according to international standards—as the minimum intensity of the magnetic field able to generate a MEP of at least 50 μV in amplitude in approximately 50% of 10 consecutive stimuli (Rossini et al., 2015, 2019). TMS was delivered with magnetic field intensity equal to 120% of RMT. In order to record the EEG responses evoked by TMS, 120 consecutive stimuli were applied, each separated by a 6–8 s random interval with the assistance of a neuronavigator system in order to have a precise repositioning during follow‐up sessions (Softaxic Navigator System, EMS).

*Electroencephalography (EEG)* was performed with 64 recording electrodes positioned over the scalp at standardized positions according to the augmented 10–20 international system. Recordings were performed in the resting state, with the patient seated in an armchair for 5 min with the eyes open and 5 min with the eyes closed.

*Magnetic resonance imaging (MRI)* was performed using a 1.5T Philips Achieva scanner equipped with an eight‐channel SENSE head coil. Structural MRI acquisition of the entire brain volume was performed using a 3D TFE T1‐weighted sequence (TR 8.2 ms, TE 3.8 ms, FOV 240 mm, slice thickness 1 mm, FA 12°). All images were obtained using a 240 × 240 matrix with an in‐plane voxel size of 1 × 1 mm. Structural MRI acquisitions were processed using the Freesurfer image analysis suite (Fischl et al., 2002; Reuter, Schmansky, Rosas, & Fischl, 2012). Cortical reconstruction and volumetric segmentation were performed for all time points. To extract values, the intersection between gray segmentation and the ROI positioned on the hand motor area of left and right precentral gyrus were used. The hand motor cortex was identified based on its typical Ω or **ε** shape on the axial scan plane (Caulo et al., 2007). DTI acquisition involved two multishot spin‐echo EPI diffusion acquisitions with high directional resolution and gradient overplus with bhigh 700 and 1,200 (s/mm^2^), respectively. The isovoxel was 2 × 2 × 2 mm^3^ with an in‐plane FOV of 224 × 224 mm^2^ and 60 slices 2 mm in thickness with no gap. A single couple of TR and TE were set to 22,140 ms and 60 ms, respectively, as the optimal values for acquisition with bmax. Stack angles were 0° in AP and RL and 0° and 15° in FH. For each session, DTI acquisitions were denoised (Manjón et al., 2013), and then, eddy current correction was applied. Second acquisition was rotated to the first one (Lotze, Flor, Grodd, Larbig, & Birbaumer, 2001) and then concatenated to get a 64 gradient direction.

*Functional MRI (fMRI)* acquisitions (task and rest acquisitions) were obtained using a gradient‐echo EPI sequence to measure the BOLD contrast over the whole brain (TR = 1,710 ms, TE = 30 ms, 34 slices acquired in ascending interleaved order, voxel size = 3.59 × 3.59 × 3.59 mm, 64 × 64 matrix, flip angle = 70°). Task for fMRI acquisition included the following: a paradigm similar to the one adopted by Macuga and Frey (2012) was performed and involved kinesthetic imagery and synchronous imitation of hand (finger tapping) movements under controlled visual drive for movement characteristics. The hand runs included two repetitions of three conditions (contralesional hand movement imitation, contra‐ and ipsi‐lesional hand imagery) presented in random order. Each condition included 2 s. of instruction followed by 16 s. of task execution and 12 s. of rest. Resting‐state functional acquisition consisted of a single run of 170 functional volumes with the same characteristics of task acquisition. During the acquisition, the patient was asked to stay relaxed and with closed eyes.

### 2.3 Data analysis

#### 2.3.1 EEG

EEG data were processed in Matlab R2014b using scripts based on the EEGLAB toolbox (Swartz Center for Computational Neurosciences). EEG signals were band‐pass filtered from 0.1 to 47 Hz using a finite impulse response (FIR) filter. Imported data were divided into 2 s duration epochs, and visible artefacts in the EEG recordings were removed using an independent component analysis (Infomax ICA algorithm) procedure (Hoffmann & Falkenstein, 2008; Jung et al., 2001). The three‐dimensional distribution of the EEG activity was estimated by the use of the *standardized low‐resolution electromagnetic tomography algorithm* (sLORETA). All EEG data epochs were normalized and recomputed into cortical current density time series at 6,239 cortical voxels. The voxels were collapsed at six regions of interest (ROI) in each hemisphere, central (Brodmann areas: 8–11 and 44–47), frontal (BA: 1–4 and 6), parietal (BA: 5–7, 30, 39–40 and 43), occipital (BA: 17–19), temporal (BA: 20–22, 37–38, 41–42), and limbic (BA: 31–36) coded according to the Talairach space.

Two different statistical analyses were performed: the first on the sLORETA intracranical power density values computed in the 12 ROIs, and the second on the graph analysis patterns extracted with eLORETA from the 84 ROIs (42 ROIs for the left and 42 ROIs for the right hemisphere). sLORETA statistical analysis was computed between the power density values of the EEG cortical sources in four selected factors: band (delta, theta, alpha 1, alpha 2, beta 1, beta 2, and gamma), side (left and right hemispheres), ROI (central, frontal, parietal, occipital, temporal, and limbic), and condition (baseline or T0, T1). The second analysis included three ANOVAs performed on the graph analysis patterns, that is, characteristic path length, clustering coefficient, and *small‐world* index and for the factors band, side, and condition. The *Greenhouse & Geisser* correction was used for protection against the violation of the sphericity assumption in the repeated measures ANOVA. Additionally, post hoc analysis was performed with Duncan's test at a significance level of 0.05. All the statistical analysis was performed using Statistica v.7 (StatSoft Inc.). The normality of the data was tested using the Kolmogorov–Smirnov test, and the hypothesis of Gaussianity could not be rejected. The significance level was set at *p* < .05.

Brain connectivity was computed by eLORETA software on 84 ROIs defined according to the 42 BAs for the left and 42 BAs for the right hemisphere. Among the eLORETA current density time series of the 84 ROIs, intracortical lagged linear coherence was computed between all possible pairs of the 42 ROIs for each of seven EEG frequency bands (delta (2–4 Hz), theta (4–8 Hz), alpha 1 (8–10.5 Hz), alpha 2 (10.5–13 Hz), beta 1 (13–20 Hz), beta 2 (20–30 Hz), and gamma (30–45 Hz)) in each hemisphere. The values of connectivity computed between all pairs of ROIs for each frequency band were used as a measure of the weight of the graph in the graph analyses. Two core measures of graph analysis were computed: characteristic path length (L) and clustering coefficient (C), representative of network global and local interconnectedness, respectively (Watts & Strogatz, 1998). *Small‐worldness* (SW) was obtained by the ratio between normalized C and L, as it describes the balance between local connectedness and global integration of a network.

#### 2.3.2 TMS‐EEG

TMS‐EEG data were processed offline using the Brain Vision Analyzer (Brain Products GmbH) and the MATLAB environment (MathWorks Inc.).

TMS‐evoked EEG activity was visually inspected in each channel, and trials contaminated by environmental artifacts, muscle activity, or eye movements were rejected. As a first step, a linear interpolation from 1 ms before to 10 ms after the TMS pulse was applied to remove the TMS artifact. Afterward, the signal was band‐pass filtered between 1 and 80 Hz (Butterworth zero‐phase filters). A 50 Hz notch filter was also applied to reduce noise from electrical sources. Identification and removal of artifacts (muscle activity, eye movements, and blink‐related activity) and TMS‐related artefactual components were done with independent component analysis (INFOMAX‐ICA). To evaluate changes in cortical excitability, both global and local cortical responses evoked by TMS were taken into account: TMS‐evoked potentials were recorded at all electrodes, and EEG responses were recorded at electrodes close to the stimulation site (Bonato, Miniussi, & Rossini, 2006; De Gennaro et al., 2008).

TMS‐EEG responses over all the included trials for each electrode and each subject were averaged in the whole epoch from 100 before to 500 ms after single TMS pulse, for each time‐point of evaluation and stimulated area. All epochs were baseline corrected to a time period of 100 ms recorded before TMS pulse (Pellicciari, Veniero, & Miniussi, 2017). Furthermore, the time domain was investigated and it was calculated the total brain activation evoked by TMS in each hemisphere (right and left) and in each condition (baseline and T1) by means of the global‐mean field power (GMFP). The GMFP is a measure of global brain activation calculated as the root mean squared value of the EEG signal across all electrodes (Lehmann & Skrandies, 1980). Then, for the analysis of the evoked responses, averaged TMS‐EEG responses over all the included trials for each electrode and each subject were used (Ferreri et al., 2011).

#### 2.3.3 MRI

Diffusion Toolkit and Trackvis (Wedeen et al., 2008) were used to calculate the diffusion tensor and fiber tracking of the corticospinal tract. The multiple volume DWI acquisitions were first corrected for eddy current and motion artifact by eddy_correct, then analyzed to get whole brain indexes values. Fraction anisotropy (FA) was the main output from the FSL Diffusion Toolkit (FDT, 24).

Cortical reconstruction and volumetric segmentation were performed with the Freesurfer image analysis suite, which is documented and freely available for download online (http://surfer.nmr.mgh.harvard.edu/). fMRI data were preprocessed with a standard procedure by using FSL toolbox (Jenkinson, Beckmann, Behrens, Woolrich, & Smith, 2012; Smith et al., 2004; Woolrich et al., 2009; Woolrich, Ripley, Brady, & Smith, 2001).

The first Feat level analysis (Woolrich et al., 2001) was performed to get functional activation on motor and imagery task. Stimuli were presented electronically using the E‐Prime 2.0 software (Psychology Software Tools); then, the “.prt” stimuli text files were converted in events (“.evt”) FSL text format by hand. The general linear model from Feat indicates the brain activities during tasks.

Resting‐state fMRI. Functional connectivity was assessed by REST time–course extraction from studied (Shirer, Ryali, Rykhlevskaia, Menon, & Greicius, 2012). ROI in Sensorimotor and DMN Networks after applying the inversion of combination between affine matrices, the first one from affine registration between BOLD and anatomical subject subspaces and the second one with atlas anatomical space. Correlations in ROIs time–course were used to build up the functional connectivity matrix (Bastos & Schoffelen, 2015).

## 3 RESULTS

### 3.1 TMS‐EEG

**FIGURE 2:**
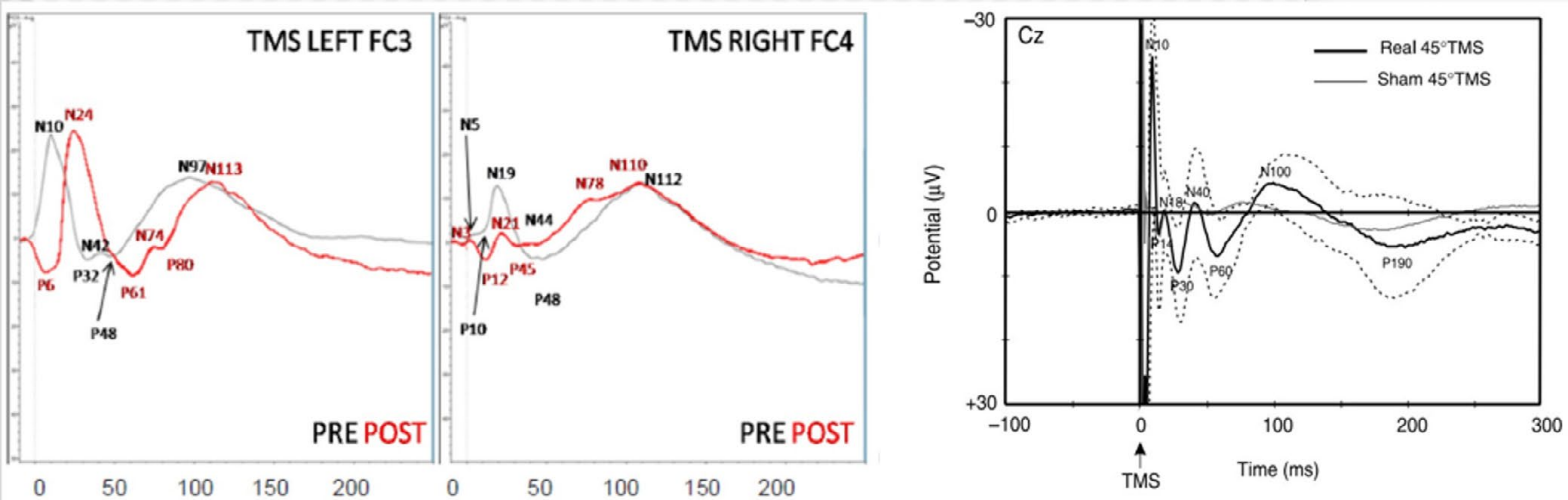
Left panel: Grand average of the TMS‐EEG responses recorded at the electrode FC3 for the left hemisphere at the baseline (black line) and T1 (red line). Middle panel: Grand average of TMS‐EEG responses recorded at the electrode FC4 in single‐pulse stimulation for the right hemisphere at baseline (black line) and T1 (red line). Right panel: trace of a normal subject (courtesy of Bonato et al., 2006). The EEG responses of left hemisphere (the spared one since the patients were all left amputees) are similar to a normal subject. The early components of EEG responses, within 50 ms, of right hemisphere (the contralateral to the amputation) are less represented and smaller in amplitude

The TMS‐EEG performed by stimulating the hot spot motor area of either the biceps or forearm flexors–extensor muscles showed a visually interhemispheric difference at baseline (T0 = before trial). Stimulation of the M1 cortex contralateral to the stump showed a visually remarkable amplitude reduction of the early components (from 10 to 50 ms poststimulus latency), while there were no significant differences in later ones (from 100 ms onward). At T1 (following the trial with the prosthetic hand), stimulation of M1 to the intact hand showed no visual significant changes in the main early and late TMS induced waves (Figure 2), while a significant modulation (*t* test, *p* < .0001) in the amplitude of the wavelets between the 30 and 100 ms poststimulus epochs was recorded for stimulation of M1 to the stump, with a significant reduction in global cortical excitability in the stimulated hemisphere, but also in the one ipsilateral to the stump.

In summary, the qualitative evaluation (visual inspection) of TMS‐EEG assessment, not supported by statistical analysis, showed at baseline in almost all patients a poor representation of the early TMS evoked potentials after M1 stimulation contralateral to the amputation, with a clear interhemispheric asymmetry that was not clearly modified at T1.

### 3.2 EEG

**FIGURE 3:**
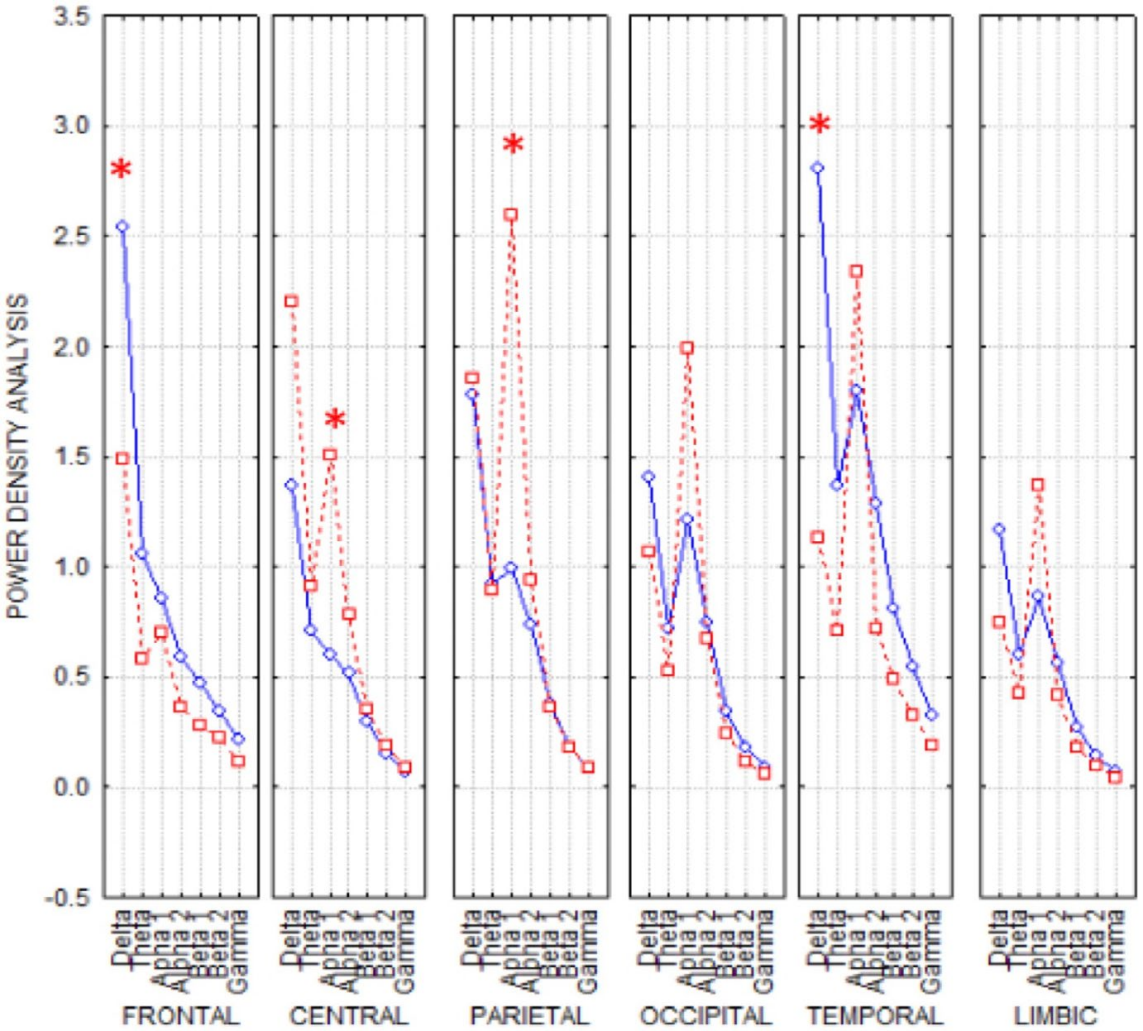
ANOVA interaction: *F* (30,210) 5 *p* < .077) of the power density values among the factors ROIs (frontal, central, parietal, occipital, temporal, limbic), Band [delta (2–4 Hz), theta (4–8 Hz), alpha 1 (8–10.5 Hz), alpha 2 (10.5–13 Hz), beta 1 (13–20 Hz), beta 2 (20–30 Hz), and gamma (30–40 Hz)], and condition (baseline and T1). Duncan‐planned post hoc testing showed that the pattern baseline > T1 was fitted in the delta band in frontal (*p* < .032206) and temporal regions (*p* < .001632) while the opposite trend baseline < T1 was found in alpha band in central (*p* < .066) and parietal (*p* < .002682) regions (asterisks for significant difference)

The ANOVA analysis for the evaluation of EEG power density values of both hemispheres (Figure 3) showed a reduction in the delta band at T1 with respect to baseline in the frontal and temporal areas while central and parietal regions presented a significant increase in alpha activity at T1 with respect to baseline. In fact, the Duncan‐planned post hoc *baseline* versus *T1* test showed lower values in the delta band in the frontal (*p* < .032206) and temporal regions (*p* < .001632), and an increase in alpha‐band activity in the central (*p* < .066) and parietal (*p* < .002682) one.

**FIGURE 4:**
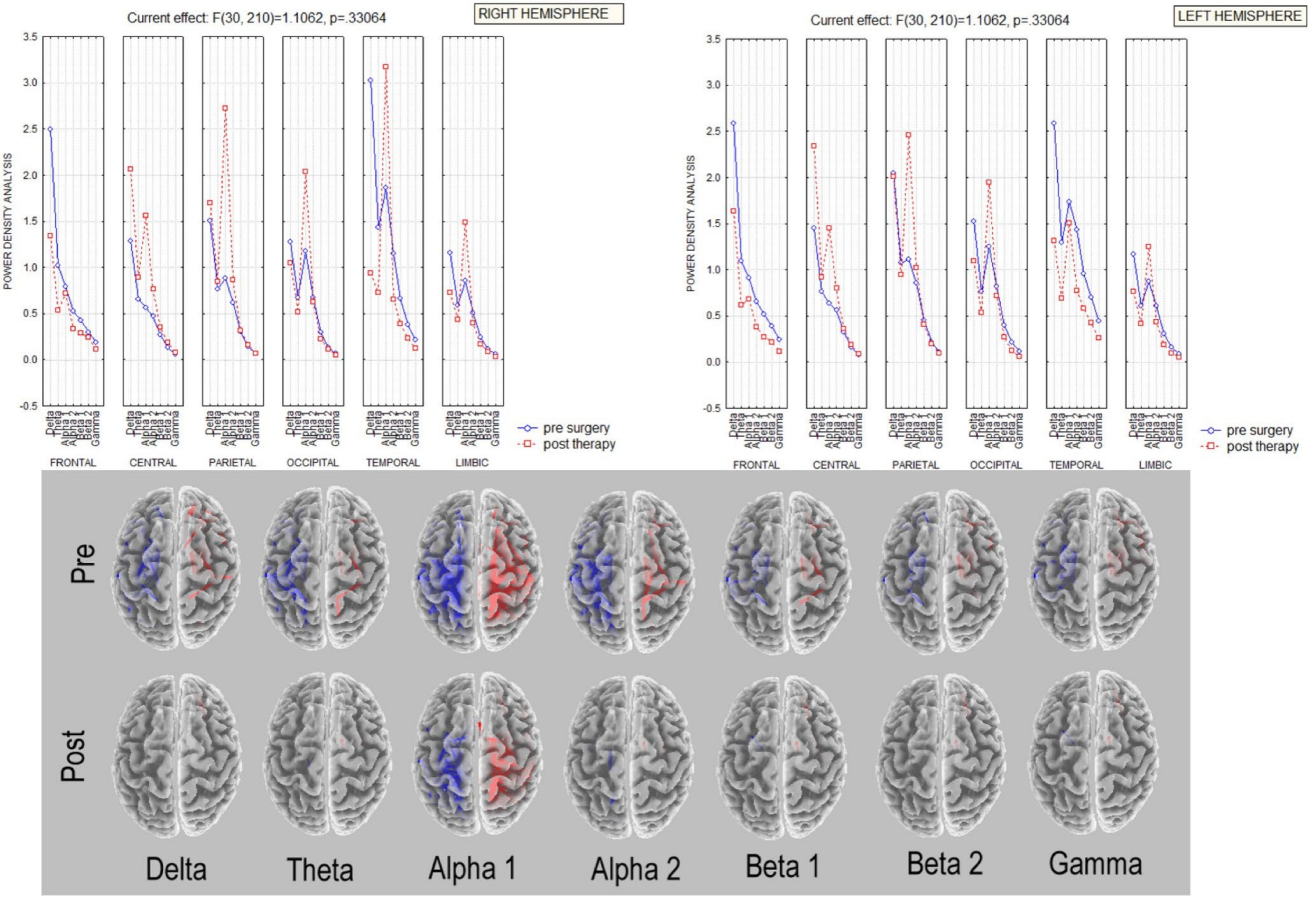
EEG power density of right and left hemispheres. Upper Left and right panels: ANOVA interaction of the power density values among the factors ROIs (frontal, central, parietal, occipital, temporal, limbic), Band (delta, theta, alpha 1, alpha 2, beta 1, beta 2 and gamma), and condition (baseline; T1) in the left hemisphere (left panel) and in the right hemisphere (right panel). Lower panel: lagged linear baseline versus T1 connectivity in EEG bands (delta, theta, alpha 1, alpha 2, beta 1, beta 2 and gamma) in the left (blue lines) and in the right hemisphere (red lines)

**FIGURE 5:**
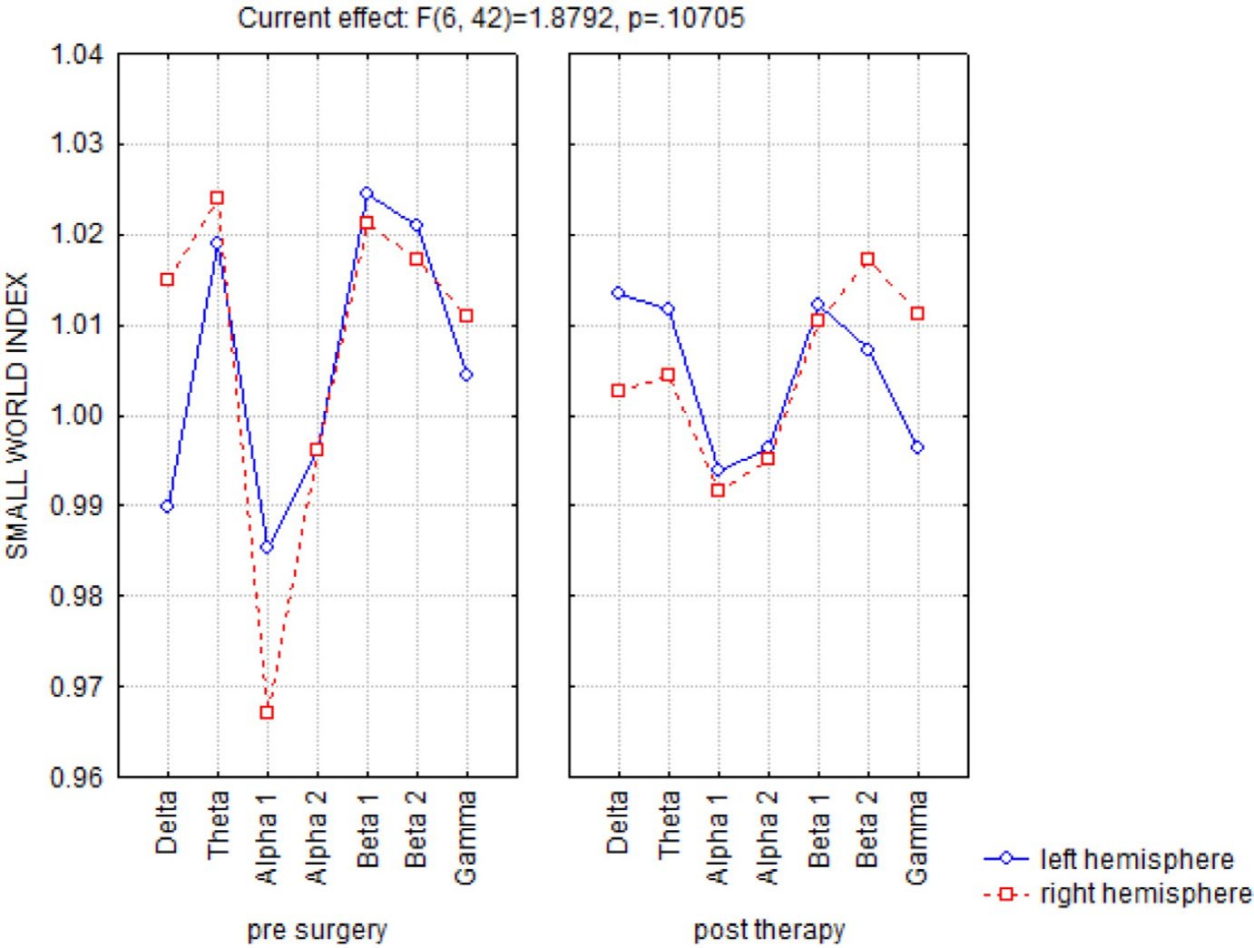
Small‐word index of right and left hemispheres at baseline and T1. ANOVA interaction of the small‐world index among the factors Band [delta (0.5–4.5 Hz), theta (5–7.5 Hz), alpha (8–11.5 Hz), sigma (12–15.5 Hz), and beta (16–24.5 Hz)] and side (left hemisphere, right hemisphere) at baseline (left panel) and T1 (right panel). After robotic hand use and sensory feedback trials, the two hemispheres appeared less different in small‐worldness

Looking at the two hemispheres separately, a nonsignificant interaction was observed between the factors band (delta, theta, alpha 1, alpha 2, beta 1, beta 2, and gamma), ROI (regions of interest: frontal, central, parietal, occipital, temporal, and limbic), and condition (baseline vs. T1) in both hemispheres, but only a trend for significance of the delta and alpha bands (Figure 4). Clustering coefficient and characteristic path length connectivity parameters were not significantly different in the same ANOVAs. The small‐world index results in baseline showed a trend (nonstatistically significant) to more random architecture in the alpha band and the opposite behavior in the delta band on the left (intact hand) with respect to the right (contralateral to stump) hemisphere. After hand prosthesis use and sensory feedback trials (T1 session), the two hemispheres showed decreased differences in *small‐worldness* for both delta and alpha bands with respect to baseline (Figure 5).

In summary, EEG recordings showed a bilateral statistically significant increase in alpha‐band power and a consensual decrease in delta band power between T0 and T1. All the other parameters analyzed did not reach the statistical significance, but for some parameters a trend for significance comparing the two hemispheres was found.

#### 3.2.1 MRI

**FIGURE 6:**
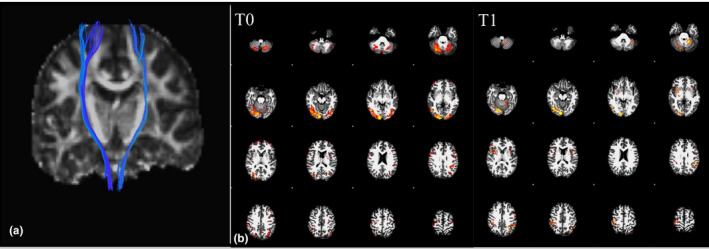
(a) MRI tractography. Corticospinal tract reconstruction. No statistically significant changes between the two sides were observes. (b) Functional MRI. Brain activation during movement imagination task of the phantom hand before and after the training. The upper panel shows the cerebral activation during the task at baseline, while the lower panel shows the activation at the end of the trial. Activation of the occipital areas is due to visual stimulation by the virtual hand movements in a screen. Following the training trials (T1), there was a reduction of additional motor areas and cerebellum activation with a prominent and more selective activation of the contralateral M1 as a typical sign of “motor learning.” Activation maps are displayed with the same statistical threshold

*DTI*. No significant differences in the fractional anisotropy of the corticospinal tracts were found between baseline and T1 (Figure 6a).

Cortical thickness. The cortical thickness of S1 and M1 remained unchanged in the baseline versus T1 images.

Task‐based fMRI. Experiments were the only task‐related ones being performed during kinesthetic imagery and synchronous mental imitation of hand movements (rhythmic finger–thumb opposition movement) under controlled visual stimulation. Baseline (T0) fMRI obtained during imagination of phantom hand movement demonstrated activation of the contralateral M1 and the cerebellum. Following training (T1), the same task demonstrated a reduction of cerebellum activation and an increase of contralateral M1 activation (Figure 6b).

*Resting‐state fMRI*. The analysis of the resting state showed a reduction in the functional connectivity on the sensorimotor network (SMN)—that is, *patient 3* from 0.38477 to 0.25781 and *patient 5* from 0.42779 to 0.35449—without changes in functional connectivity within the default mode network (DMN; i.e., *patient 3* from 0.21194 to 0.18664 and *patient 5* from 0.26041 to 0.25376).

## 4 DISCUSSION

Our study provides a direct evidence of brain functional changes (as addressed via different techniques) following training with bidirectional hand prosthesis in five amputees; two of them had a relatively short‐term trial (of 1 month) while the others had a longer training period (5, 6, and 9 months). All subjects had suffered from a left transradial amputation and, with one exception, were right‐handed. Background conditions were somewhat different considering the duration of amputation, the presence and intensity of phantom limb pain (PLP), and the use of the commercially available prosthesis before the trial.

It should be taken into account that our primary endpoint was to reveal chronic and stable changes in the functional organization of the sensorimotor brain areas due to amputation, either contralateral to amputation or in both hemispheres, and to follow up their eventual modifications induced by training with the sensorized hand prosthesis of last prototypal generation. For this reason, most of the investigations were carried out under resting conditions and not during a task.

EEG recordings showed a bilateral statistically significant increase in alpha‐band power and a consensual decrease in delta band power. The separate analysis of the two hemispheres did not show a statistically significant change in these two EEG frequency bands, with only a trend toward significance. EEG resting‐state connectivity showed baseline asymmetry of the two hemispheres, especially in the alpha and delta bands, with a tendency (even in this case nonstatistically significant) toward a more symmetrical representation after training with the bidirectional hand prosthesis in the T1 recordings. Even if many of our data showed only a trend of significance, probably because of the reduced number of patients, some consideration could be made. Alpha rhythms reflect one of the most prominent oscillatory hallmarks of the resting/awake human brain and may arise from different cortico–cortical and cortico–thalamic–cortical circuits that play an important role in the top–down control of cortical activation (Palva and Palva, 2011) and exhibit temporal correlations and spatial coherence over a long time range (Freyer, Aquino, Robinson, Ritter, Breakspear, 2009; Nunez, Wingeier, Silberstein, 2001). Conversely, the localized delta frequency band in awake adults is usually associated with focal cerebral dysfunctions/lesions. Moreover, a generalized reduction in alpha EEG and a simultaneous increase in delta rhythms are a common pattern of progressive brain diseases or loss of sensory information, for example, cognitive impairment and chronic visual deprivation (Bola et al., 2014; Vecchio et al., 2014). Recovery of the alpha/delta balance, that is, an increase in alpha activity and a consensual decrease in slow rhythms in the delta range, has been described following clinical improvement (Bola et al., 2014) that, in the case of hand amputation, could be considered as regaining a “surrogate” of the lost function via an artificial hand (movement and sensory feedback). Interestingly, the analysis of EEG connectivity showed a trend toward a focal effect predominantly on the hemisphere controlling the amputated hand. It can therefore be hypothesized that the use of a last‐generation prosthesis closely reproducing the motor/sensory conditions of the natural hand has different effects on brain EEG rhythms, that is, some more diffuse and bihemispheric, others more focal and monohemispheric, but specifically related to the input/output of the M1 and S1 areas contralateral to the prosthesis. This information supports our hypothesis that the regular use of a prosthetic hand mimicking a "natural" hand activity could reverse some of the aberrant plastic changes following amputation.

Structural MRI did not show significant modifications in corticospinal tract fiber density or in S1‐M1 cortex thickness neither at baseline, nor after training, suggesting that the observed brain changes were mainly due to synaptic strength and connectivity changes without significant anatomical modifications. The reason for this could be related to several factors including the relatively short duration of the trials and the use of the prosthetic hand mainly in a laboratory context more than in an ecological environment. Moreover, the connectivity analysis performed with the fMRI data allowed examining the changes occurring in two networks with different functional roles, that is, the SMN and the DMN. We found significant modifications in connectivity mainly restricted to the SMN, without significant changes in the DMN in line with previous results (Makin, Filippini, et al., 2015); this supports the idea that, after amputation, the missing hand cortex gradually becomes functionally decoupled from the SMN, its typical network of origin, and that prolonged use of a sensorized hand prosthesis could reverse this phenomenon. Moreover, when fMRI findings were followed up during a task with phantom hand movement (repeated movements opening and closing), an increase of M1 and a decrease of cerebellar recruitment was found at follow‐up, supporting the idea that the prolonged use of the bidirectional prosthetic hand can reverse some aberrant brain plastic changes. These findings, even if at some difference, could be considered similar to those of recent studies exploring brain changes after restoration of bidirectional hand functionality after amputation. Indeed, Valyear et al. (2019) showed with fMRI in a patient with hand transplantation an increasing activation of brain areas involved during grasping (mainly the anterior intraparietal cortex, premotor and cerebellar cortices) over time, with a final condition more similar to controls subjects. Moreover, Serino et al. (2017) demonstrated in amputees with targeted muscle and sensory reinnervation (TMSR) how primary motor and sensory cortices activity related to movement and touch are enabled by targeted muscle and sensory reinnervation, suggesting that TMSR may counteract maladaptive cortical plasticity typically found after limb loss, in M1, partially in S1, and in their mutual connectivity.

TMS‐EEG findings, even if not supported by statistical analysis, showed at baseline recordings a poor representation of the early TMS evoked potentials after M1 stimulation contralateral to the amputation, with a clear interhemispheric asymmetry. Interestingly, this phenomenon was evident almost exclusively for the early waves (before 50 ms) that are related to the direct activation of the stimulated motor cortex and the spread of activation to the contralateral hemisphere via direct transcallosal (P14) and/or deep subcortical connections (wave P30 as seen in the paper by Bonato et al. (2006)). There was no significant modification of these waves after training, suggesting that the use of a prosthetic system was not able to reverse some of the alterations in cortico–cortical connectivity following amputation.

Modifications of the PLP are an indirect, but still powerful reflection of the functional changes described in this study. Four out of five patients suffered from PLP, and three of them improved following training with the prosthesis. *Patient 1* experienced a 4–2 decrease in PLP score on the visual analogic scale (VAS), while *patient 2* decreased from 9 to 4 and *patient 3* from 8 to 4. *Patient 5* did not experience any PLP modification during the trial, with a stable VAS score of 4 (see Table 1).

Our study is affected by several limitations: (a) The methodology was not homogeneous due to patient characteristics, patients availability to be submitted to several investigations and regulatory requirements (i.e., two of them could not undergo MRI due to claustrophobia; in one, the type of intraneural electrodes used was not compatible with use in the MRI environment); (b) the prosthetic system was not exactly the same; (c) the duration of the trial was different across patients due to health authorities restrictions; and (d) the absence of a control group.

Moreover, the influence of the different clinical conditions of the patients at baseline should be also considered, especially the role of time since the amputation and the presence/absence of PLP. The low number of patients combined with the just mentioned limitations does not allow more complex conclusions that would result in excessive speculation. Despite such limitations, this study describes a unique and unprecedent experience accumulated on this topic in 10 years, clearly showing that brain reactions to multitask (motor and sensory) artificial hand use can be tracked for a tailored approach to a fully embedded artificial upper limb for future chronic uses in daily activities.

## 5 CONCLUSIONS

Our study provides a direct evidence of brain functional changes (as addressed via different techniques) following training with bidirectional hand prosthesis in five amputees. The alterations, describing for the first time the brain reactions to multitask (motor and sensory) artificial hand use, were mainly restricted to functional connectivity as addressed with EEG, TMS‐EEG, and MRI and could represent a starting point for a future tailored approach to study chronic uses of fully embedded artificial upper limb in daily activities.

## CONFLICT OF INTEREST

The authors declare no competing interest.

## AUTHOR CONTRIBUTION

G.G. and P.M.R. conceived the study, planned the experiments, and wrote the paper. F.M. analyzed data on EEG and brain connectivity and wrote the paper. M.C. analyzed MRI data and wrote the paper. R.D. and F.Io. performed the experiments, collected clinical data, and drafted the final version of the paper. F.V. analyzed data on EEG and brain connectivity. G.V., I.S., E.D., and F.Ib. performed the experiment. L.L. and E.F. performed surgical implantation of the electrodes. R.R. performed the experiments and drafted the paper. F.M.P., S.R., and S.M. conceived the study and drafted the paper.

### Peer Review

The peer review history for this article is available at https://publons.com/publon/10.1002/brb3.1734.

## Data Availability

The data that support the findings of this study are available on request from the corresponding author. The data are not publicly available due to privacy or ethical restrictions.
